# A randomized controlled trial of a multi-modal palliative care intervention to promote advance care planning and psychological well-being among adults with advanced cancer: study protocol

**DOI:** 10.1186/s12904-022-01087-z

**Published:** 2022-11-17

**Authors:** Joanna J. Arch, Jill L. Mitchell, Sarah J. Schmiege, Michael E. Levin, Sarah R. Genung, Madeline S. Nealis, Regina M. Fink, Emma E. Bright, David J. Andorsky, Jean S. Kutner

**Affiliations:** 1 Department of Psychology and Neuroscience, University of Colorado Boulder, 345 UCB Muenzinger, 80309-0345 Boulder, CO USA; 2 Division of Cancer Prevention and Control, University of Colorado Cancer Center, 80045 Aurora, CO USA; 3 Rocky Mountain Cancer Centers, 80111 Greenwood Village, CO USA; 4 Department of Biostatistics and Informatics, Colorado School of Public Health, University of Colorado Anschutz Medical Center, 80045 Aurora, CO USA; 5 Department of Psychology, Utah State University, 84322 Logan, UT USA; 6 University of Colorado College of Nursing, Anschutz Medical Campus, 80045 Aurora, CO USA; 7 Division of General Internal Medicine, Department of Medicine, University of Colorado School of Medicine, Anschutz Medical Campus, 80045 Aurora, CO USA

**Keywords:** Palliative care, cancer, Anxiety, Depression, Advance care planning, Acceptance and commitment therapy

## Abstract

**Background:**

Up to half of adults with advanced cancer report anxiety or depression symptoms, which can cause avoidance of future planning. We present a study protocol for an innovative, remotely-delivered, acceptance-based, multi-modal palliative care intervention that addresses advance care planning (ACP) and unmet psychological needs commonly experienced by adults with metastatic cancer.

**Methods:**

A two-armed, prospective randomized controlled trial (RCT) randomizes 240 adults with Stage IV (and select Stage III) solid tumor cancer who report moderate to high anxiety or depression symptoms to either the multi-modal intervention or usual care. The intervention comprises five weekly two-hour group sessions (plus a booster session one month later) delivered via video conferencing, with online self-paced modules and check-ins completed between the group sessions. Intervention content is based on Acceptance and Commitment Therapy (ACT), an acceptance, mindfulness, and values-based model. Participants are recruited from a network of community cancer care clinics, with group sessions led by the network’s oncology clinical social workers. Participants are assessed at baseline, mid-intervention, post-intervention, and 2-month follow-up. The primary outcome is ACP completion; secondary outcomes include anxiety and depression symptoms, fear of dying, and sense of life meaning. Relationships between anxiety/depression symptoms and ACP will be evaluated cross-sectionally and longitudinally and theory-based putative mediators will be examined.

**Discussion:**

Among adults with advanced cancer in community oncology settings, this RCT will provide evidence regarding the efficacy of the group ACT intervention on ACP and psychosocial outcomes as well as examine the relationship between ACP and anxiety/ depression symptoms. This trial aims to advance palliative care science and inform clinical practice.

**Trial Registration:**

Clinicaltrials.gov NCT04773639 on February 26, 2021.

## Background

The Institute of Medicine’s “Dying in America” report [1] and the National Institutes of Health [2] identify the palliative care needs of the growing population of adults with life-limiting disease as an urgent health care priority. There is a vital need for novel palliative care interventions that are effective, operate through clear testable mechanisms, can be personalized to meet individual needs, reach patients with limited access to palliative care specialists, and inform health care practice. This paper presents a protocol for a randomized controlled trial (RCT) to evaluate a novel primary palliative care intervention for anxious and depressed adults with advanced cancer (i.e., adults with metastatic disease or similar prognosis) that aims to progress the science to address these priorities. The intervention is a multi-modal intervention based on acceptance and commitment therapy (M-ACT) that was originally designed to be delivered via in-person group sessions alternating with online, self-paced modules and check-ins to deepen understanding of skills learned to apply them in daily life. However, the trial received funding at the beginning of the COVID-19 pandemic and consequently, prior to enrolling any participants, was adapted to be conducted entirely remotely. The resulting protocol thus addresses the need for palliative care interventions that can be delivered remotely, which offers the twin advantages of increasing scalability and reaching patients isolated by distance, travel, or health restrictions. This and other pandemic-related changes follow in concordance with the SPIRIT-CONSERVE guidelines [3].

Adults with advanced cancer report a range of palliative care challenges, including anxiety and depression in the face of imminent loss and how to make the most of their remaining time, spiritual/ existential needs related to making sense of their illness, fear of dying/death, and physical symptoms [4–11]. To engage themselves and their loved ones in advanced cancer-related care and decision making, many recommend that patients engage in advance care planning (ACP), defined as “a process that supports adults at any age or stage of health in understanding and sharing their personal values, life goals, and preferences regarding future medical care” [12]. Many palliative care interventions address a single dimension of palliative care – e.g., ACP directive completion or sense of life meaning – or focus on patients in general rather than the anxious and depressed patients most in need of support [5, 13–15]. Up to half of adults with metastatic cancer report elevated anxiety or depression [9, 16, 17], which can be associated with poor quality of life, more severe physical symptoms [18, 19], and earlier cancer-related mortality [20–22]. Further, many interventions for adults with metastatic cancer (e.g., [14, 23]) are disconnected from the scientific evidence base on how to effectively reduce distress or change behavior. The M-ACT intervention reflects a paradigm shift in palliative care by leveraging ACT [24]–an evidence-based approach for reducing distress and changing behavior–to address the palliative care needs of psychologically vulnerable patients with advanced cancer by reducing avoidance of internal experience (e.g., feelings/thoughts) [25] and increasing sense of meaning and values-aligned behavior change. Thus, M-ACT differs from general ACT protocols in two dimensions: first, through its multi-modal delivery and second, through its adaptation to address the ACP and psychological needs of adults with advanced cancer.

Though notable meaning-focused interventions for metastatic cancer patients exist (e.g., [14, 15]), none directly target avoidance of internal experience [25], a process underlying the psychological disorders most common among cancer patients [26, 27]. By aiming to increase life meaning *and* reduce internal avoidance, M-ACT represents a new and potentially more powerful intervention for anxious and depressed adults with metastatic cancer. In contrast to behavioral interventions that focus on mastery and control of distress, M-ACT *allows for* distress to help people live meaningfully no matter what their circumstances – an approach particularly well suited for patients facing incurable disease [25, 28]. By facilitating meaningful living in the face of uncertainty, M-ACT holds promise for helping those with metastatic cancer to reduce anxiety and depression. By helping patients to reflect on their values and make decisions aligned with these values, M-ACT holds similar promise as an approach to ACP. Finally, based on the broader ACT model, M-ACT specifies putative mechanisms of change including increasing active acceptance and reducing avoidance of internal experiences (i.e., difficult thoughts and emotions) and clarifying values and sources of meaning (e.g., [29–31]), mechanisms shown to predict psychosocial outcomes in our previous ACT studies [32, 33].

Most cancer patients in the United States (US) receive care from community-based oncology pratices [34]. These clinics often lack access to palliative care specialists [35, 36], which presents barriers to addressing patients’ palliative care needs [37]. Palliative care research has developed personally tailored, individually delivered interventions requiring significant resources (e.g., [14, 23, 38]) or group programs that are more efficient but require standard, less personalized intervention content across diverse participants [39]. Online-only interventions without provider support also reduce resource demands but result in poor completion rates [40] and often limited effects on the outcomes of current interest [4]. In contrast, M-ACT combines the best of group-based and online delivery to offer personalized and efficient primary palliative care. M-ACT’s professionally-facilitated group sessions aim to increase intervention efficacy by supporting acquisition of skills and increasing accountability for completing the online self-paced modules and check-ins done between group sessions [41]. These self-paced components (the online modules and check-ins) further personalize the intervention by tailoring online content to individual needs without increasing demand on providers.

Finally, given the nationwide shortage of palliative care specialists [35, 36], approaches that do not demand a specialized resource in short supply are needed to increase the accessibility and reach of palliative care. We designed M-ACT for settings that lack onsite palliative care specialists. Further, we designed M-ACT for delivery by community-based oncology clinical social workers in a manner that is consonant with their existing roles (e.g., to facilitate cancer support groups).

### The M-ACT pilot studies

We conducted a single-arm pilot trial (*n* = 35) in which we developed, refined, and preliminarily tested a beta version of M-ACT collaboratively with community oncology social workers. We rigorously refined this beta version in response to participant and provider feedback and conducted usability testing. We also evaluated its initial efficacy potential among anxious and depressed adults with metastatic cancer [42]. This pilot trial resulted in a refined M-ACT facilitator manual for the group sessions and user-friendly online sessions and daily check-ins, ready for testing in a RCT. We also demonstrated the feasibility and acceptability of the intervention, with strong study enrollment and session attendance rates. In terms of efficacy potential, M-ACT in the pilot trial was associated with significant improvements from baseline to post-intervention as well as 2-month follow-up on ACP engagement, anxiety and depression symptoms, and acceptance and sense of meaning.

In response to the COVID-19 pandemic, the M-ACT group sessions were adapted from in-person group delivery to online videoconferencing. Online delivery of group interventions has shown good feasibility in previous studies (e.g., [43]). Nonetheless, to help ensure feasibility and acceptability of shifting the M-ACT group sessions online, we first adapted the M-ACT facilitator manual to the online environment. We tested this adapted manual within a second, single-arm pilot study with eight anxious and depressed adults with metastatic cancer to solicit extensive qualitative and quantitative participant feedback and further refine the facilitator manual prior to its evaluation in the current trial. Two significant changes were made to the study protocol during this process, including adding an online group booster session one month after the final weekly group session, which pilot participants requested to help them recall and use the skills taught in the online group, and simplifying and clarifying the items on the ACP Checklist that serves as this trial’s primary outcome (see Measures).

### The current study protocol

This protocol describes an RCT to evaluate the efficacy of the adapted M-ACT intervention designed to increase ACP and address the psychological needs of anxious and depressed adults with metastatic cancer. This trial addresess a number of limitations in previous trials of palliative care interventions by: (1) adapting an evidence-based intervention model (ACT) for adults with advanced cancer, (2) evaluating theory-driven, testable mechanisms of the intervention, (3) intervening at the group level yet adding self-paced online components that personalize the intervention to individual needs, and (4) reaching patients with limited access to specialist palliative care. Finally, another major aim of this study is to evaluate the relationship between anxiety and depression symptoms, and completion of ACP—an important but underexplored relationship with the potential to inform health care practice.

M-ACT is compared to a usual care control (UC) to determine if M-ACT offers benefits beyond those realized by usual care. Thus, within a community oncology setting, this study will:

1. Evaluate the hypothesis that M-ACT, relative to UC, will increase the primary outcome of ACP (e.g., advance directive completion, values and goals of care discussions) and reduce anxiety and depression symptoms, fear of dying, and increase sense of life meaning (secondary outcomes).
2. Assess the cross-sectional and longitudinal associations between anxiety and depression symptoms and ACP, to evaluate baseline relationships as well as the longitudinal relationship between change in symptoms and change in ACP.
3. As an exploratory aim, evaluate M-ACT’s hypothesized mechanisms of active acceptance, reduced experiential avoidance, and alignment of behavior with personal values, to evaluate if they increase more following M-ACT than UC and if they predict ACP engagement and psychosocial outcomes.

## Methods/design

### Study design and funding

Our study design is a two-arm, prospective RCT that randomizes 240 anxious and depressed adults with metastatic solid tumor cancer to the M-ACT intervention or UC control condition. Participants are individually randomized 1:1 within cohorts to M-ACT or UC based on a randomization block sequence generated by the study biostatistician (S.J.S.) using SAS Version 9.4 statistical software [44]. As outlined in Table 1, participants are assessed at four timepoints: Baseline (Pre), Mid-Intervention (Mid), Post-Intervention (Post), and 2-month Follow-Up (FU), via online surveys administered in REDCap [45].

**Table 1:** Schedule of M-ACT study enrollment, intervention, and assessment

	Enrollment	Allocation	Post-allocation								
TIMEPOINT**	***PRE (-t***_***1***_ ***)***	**0**	*Group 1*	*Group 2*	*Group 3*	***MID***	*Group 4*	*Group 5*	***POST***	*Group* *Boost*	***Follow-Up (FU)***
**ENROLLMENT**:											
** Eligibility screen**	X										
** Informed consent**	X										
** Allocation**		X									
**INTERVENTION (one arm)**: **M-ACT**			X	X	X		X	X		X	
**ASSESSMENTS (both arms)**:											
*** Primary & Secondary Outcomes***	X					X			X		X
*** Process Measures***	X					X			X		X

The study was pre-registered on Clinicaltrials.gov with a first posting on February 26, 2021 (within 30 days of enrolling the first participant) under identifier NCT04773639. Study funding is provided by the National Institute of Nursing Research at the National Institutes of Health (R01NR018479) to J.J.A. This funding source had no role in study design and will not have a role in its execution, analysis, interpretation, or submission of the results. Table 2 outlines the CONSERVE strategies for each pandemic-related modification to the study protocol. As we had not yet begun the study when these pandemic modifications were made, this is Version 1 of the protocol.

**Table 2:** List of Protocol Changes Undertaken Prior to Participant Enrollment in Response to the COVID-19 Pandemic, per CONSERVE-SPIRIT guidelines

CONSERVE-SPIRIT Item/ Number	Impacts	Mitigating Strategies	Importance
9. Study setting	• Due to the pandemic, we could no longer hold the group sessions in-person onsite in cancer care clinics	• Shift group sessions from in-person to online via video conferencing	• This is a moderately important modification because it changes the physical setting in which participants join the online group sessions, which will now vary from person to person
11. Interventions	• Due to the pandemic, we could no longer hold the group sessions in-person onsite in cancer care clinics	• Shift group sessions from in-person to online via video conferencing • Add a fifth weekly group session and a booster • Adapt content delivery for online environment (use of visual slides, breakout rooms)	• These are important modifications because they change the length and delivery of the active study intervention.
13. Participant timeline	• Shift to online group session delivery increased the possibility of participant distraction and the need for extra time to deliver the same material	• Shift study participation one week longer due to addition of 5th weekly group session	• This is a minor modification, as it adds 1 additional week out of 3.5 to 4 total months of study participation.
15. Recruitment	• Could no longer recruit in-person at cancer care clinics • Did not need to limit recruitment for a given cohort to the clinic hosting the group, as group sessions moved online	• Rely more heavily on mailings and other remote recruitment methods • Due to group sessions shifting to online delivery, change recruitment for each cohort from site-specific to network-wide	• These are important modifications because they affect both the method and scope of recruitment for each study cohort.
27. Confidentiality	• Moving the groups online made it possible that non-group members (family members, roommates, etc.) could overhear the group	• Request in the first group session that participants join from a private room with a closed door or use headphones, if possible	• This is largely a minor modification reflecting the broader modification of moving the groups online
32. Informed consent materials	• The mitigation strategies listed above changed the study protocol	• Revise the consent form to reflect the changes outlined above	• These are important modifications because they inform participants that the entire active intervention will be delivered remotely as opposed to partially in-person.

### Study eligibility criteria

#### Inclusion criteria include adults (age 18+) who are

1. Diagnosed with Stage IV metastatic cancer of any solid tumor type, extensive-stage small cell lung cancer, or Stage III recurrent ovarian cancer or glioblastomas of any stage due to their aggressive nature, which is similar to Stage IV disease.
2. Capable at time of consent of understanding and voluntarily consenting themselves to the study, attending group sessions, and completing online sessions, confirmed by an Eastern Cooperative Group Performance Status Scale [46] rating of 0 to 2;
3. Endorsing moderate to severe anxiety or depression symptoms on the Patient Health Questionnaire-4 [47] (see *Screener*, below);
4. English speaking and are comfortable completing the group and surveys in English.

#### Exclusion criteria include

1. Current moderate to high suicide risk on an abridged verson of the the Columbia-suicidality rating scale interview [48];
2. Psychiatric hospitalization or suicide attempt in the past 5 years;
3. History of chronic untreated trauma (unrelated to cancer).

Adults excluded for these reasons are referred to more individualized or intensive support care resources.

### Participant recruitment and consent

Participants are recruited largely through Rocky Mountain Cancer Centers (RMCC), the largest network of community-based oncology practices in Colorado. As a result of the COVID-19 pandemic, plans to recruit in person were modified to recruit using remote methods (see Table 2). Thus, eligible patients are recruited from RMCC clinics through mailings, flyers in waiting and exam rooms, and website postings, and targeted advertising in local online and print media. We use diverse images and culturally-sensitive examples to help ensure that study recruitment materials are sensitive to racial/ ethnic minorities, reflecting the diversity of patients and providers. RMCC providers are encouraged to directly refer potentially eligible patients. As the M-ACT group sessions are led by RMCC clinical social workers (see *Study Conditions*), the social workers serve as the main direct referral source, and obtain oral consent from patients interested in the study to release their contact information to the University of Colorado research team. Participants are also recruited from other Colorado cancer care clinics as long as the treating clinic permits us to verify the participant’s medical eligibility status, though these additional participants are expected to comprise a small minority of total participants.

Once a potential participant contacts the study team for more information or is referred by a provider, the University of Colorado research team shares study details, answers questions, and screens them to determine eligibility. If the person remains interested and is eligible, the study consent process is initiated. As in our recent prior studies [49], this process is conducted remotely in three steps (1) participants are emailed or mailed (per their preference) an authorization form that provides the study team with consent to access their electronic health record in order to confirm their medical eligibility; (2) participants are emailed or mailed (per their preference) a study consent form to review on their own and given adequate time to consider; and (3) participants receive a scheduled phone call with a study team member to verbally walk through the consent form and address any questions about study participation before signing it to indicate their voluntary participation. Participants remotely sign the authorization request and consent form using secure DocuSign, a financial bank-grade secure software for managing electronic agreements. The consent form includes that study and intervention participantion is entirely voluntary and that participants can be removed at the discretion of the principle investigator (J.J.A.) for reasons such as violating reasonable expectations for study participation.

### Study conditions

Participants are recruited in cohorts that consist of an average of 10 to 12 participants, with half randomized to each condition. If patients lack a home computer, they are loaned a Wi-Fi-enabled computer tablet and keyboard for use during the study.

#### M-ACT intervention: structure and modifications

The M-ACT intervention consists of five weekly group sessions of two hours each plus an additional group booster session of the same length one month later. In between each of the first three group sessions, participants are invited to individually complete a self-paced 20–30 min online module. The three online modules review and apply in a more detailed and personally tailored manner the ACT concepts, metaphors, and skills learned in the group sessions such as responding to distress, moving toward values, and engaging in ACP. After the fourth group session, participants are once again invited to complete any online modules or review and redo any that would be helpful for re-engagement. After all group sessions, including the booster session, participants are sent a link three times a week to complete online brief check-ins that apply ACT skills learned in group to their daily lives in a visually engaging manner. M-ACT participants also receive printed workbooks with instructions for the weekly home practice, ACT worksheets, materials, and reference sheets for work done in group, select ACP forms, and symptom management tips provided by the Hospice and Palliative Nurses Association [50].

Group sessions are co-led by two RMCC clinical social workers using the detailed facilitator manual developed and refined in the original M-ACT pilot trial [42]. These sessions were originally designed to be conducted in person and were re-designed to be conducted online in response to the pandemic, using videoconferencing (Table 2). This resulted in three changes. First, the number of weekly group sessions [42] was increased from four to five to allow extra time for small group breakout sessions as the shift to an online environment necessitated slowing down presentation content, extra time to manage breakout rooms, and risked distractions from participants’ home environments. Second, the group facilitator manual was modified for the online environment in a number of ways, including by shifting presentations of new concepts to the online environment with Powerpoint slides, using online breakout rooms for smaller group work, and providing technical instructions on using Zoom to conduct an online group (Table 2). This modified manual was piloted in a single arm pilot study with eight participants and two facilitators (see *Introduction*); extensive quantitative and qualitative feedback was collected. Specifically, we received pilot participant feedback that a booster session would be helpful as participants reported having more difficulty remembering content when it was presented online, and wanted an opportunity to review it. Thus third, prior to launching the RCT, we added a booster group session one month after the fifth weekly group session.

#### M-ACT intervention: content

Table 3 briefly summarizes the group session and online module content. In the first group session, we introduce the Choice Point [51, 52] as a tool for clarifying personal values and corresponding valued actions, naming difficult internal experiences that are a struggle (thoughts, feelings, physical symptoms), and evaluating responses to those experiences that bring us closer or further away from our values (titled “Towards and Away Moves”). We introduce and define ACP, link personal values to ACP, and discuss its importance. Between group sessions 1 and 2, they are invited to complete the online Module 1 (once) and online Choice Point check-in (up to daily); both apply the Choice Point to their own life in a personalized manner.

**Table 3:** M-ACT Content Summary

Session	Content	ACT Processes Emphasized
**Group Session 1** *How do we Navigate?*	Group expectations & ground rules, Introduce Choice Point, Introduce Advance Care Planning (ACP)	Values, Valued Action, Experiential Approach/Avoidance
**Online Module 1** *Towards and Away*	Tailor Choice Point to the individual participant, Identify personal Toward and Away moves and their impact	Values, Valued Action, Experiential Approach/Avoidance
**Group Session 2** *Passengers: An Introduction*	Brief present-awareness meditation, Debrief online module 1, Introduce Passengers on the Bus metaphor, Create and share personal Passengers cards, Introduce an ACP step and encourage engagement	Mindfulness, Acceptance, Defusion
**Online Module 2** *Growing Values*	Select and learn how to appoint a healthcare proxy based on personal values and small committed actions	Acceptance, Values, Committed Action
**Group Session 3** *Practice with Passengers*	Brief present-awareness meditation, Debrief Online Module 2, Introduce defusion strategies, Do eyes-closed acceptance exercise, Experientially act out Passengers on the Bus in breakout rooms	All Hexaflex Processes (Mindfulness, Acceptance, Defusion, Self-as-Context, Values, Committed Action)
**Online Module 3** *Driving Your Bus*	Apply the Passengers metaphor to a personally challenging situation with cancer, Identify valued direction/goal and passengers that serve as barriers, Practice acceptance and defusion skills for approaching the thoughts and feelings that the Passenger triggers	All Hexaflex Processes
**Group Session 4** *Practice with Passengers 2*	Brief present-awareness meditation, Debrief Online Module 3, Eyes-closed exercise to identify and connect with a wise/kind passenger, Experientially act out Passengers on the Bus in online breakout rooms	All Hexaflex Processes
**Group Session 5** *Looking Back, Looking Ahead*	Brief present-awareness meditation, Reflect and review content/skills learned in M-ACT, Coach self to use skills in future via writing letter to self (mailed 3 weeks later), Identify skills to continue using, Do lovingkindness meditation	All Hexaflex Processes
**Group Booster Session** *Reconnect*	Brief present-awareness meditation, Check-in, Review and troubleshoot use of M-ACT skills in daily life, Commit to ‘next best move’ in ACP/cancer	All Hexaflex Processes
**Brief Online Check-Ins**		
**Check-In 1** *Choice Point*	Identify personal Toward moves done that day, Celebrate them, link Toward moves to values, Set new small valued goal for today or next day	Values, Committed Action, Self-as-Context
**Check-In 2** *Skills*	Identify values, Acknowledge Passengers in daily life, Practice brief interactive acceptance and defusion skills (choose from a list of each)	Values, Mindfulness, Acceptance, Defusion

Remaining group sessions begin with a brief awareness meditation to increase present-moment awareness and openness toward internal experience, followed by an opening circle in which participants debrief and share their home practice (the online modules and check-ins) for that week in two breakout rooms, with one facilitator per room. Each group session ends with introducing a new ACP step, discussing questions about it, and planning for how to engage in that step.

Each group session also offers specific M-ACT skills or metaphor foci. Session 2 focuses on introducing the *Passengers on the Bus* metaphor in the context of cancer (i.e., Passengers represent one’s most challenging thoughts and feelings in dealing with cancer, with the exercise focusing on how to skillfully respond to them while “driving” towards one’s values). Between group sessions 2 and 3, participants are invited to complete online module 2, which uses M-ACT principles as a framework for reflecting on, selecting, and communicating regularly with a health care decision maker (i.e., a health care proxy), the ACP step most emphasized in M-ACT. Between group sessions 2 and 3, participants continue to complete the brief online Choice Point check-in to maintain their connection with the Choice Point. In the second half of Sessions 3 and 4, participants experientially act out the Passengers metaphor in break-out rooms, taking turns to practice driving their bus forward in a valued direction while the other participants act as their difficult passengers, presenting the thoughts and feelings that the driver endorsed struggling with. The driver is invited to notice how they typically respond to their passengers and then is coached to use their acceptance and defusion skills to continue driving forward toward their values. Between group sessions 3 and 4, participants are invited to complete online module 3, which reinforces applying the Passengers metaphor to their own lives. This week they also are invited to shift to completing the online Skills check-in, which offers numerous cognitive defusion and acceptance exercises to practice at home. After completing online module 3, during the remainder of the program participants are invited to complete either the Choice Point or Skills online check-ins at least three times each week as well as to review or repeat any of the online modules.

The final weekly group session, Session 5, serves as a reflection and review session in which the group reviews M-ACT content and skills. The booster session that is scheduled for one month later reviews and troubleshoots their continued use of M-ACT skills in their daily lives, and ends with sharing personal commitments about their “next best move” for advancing their ACP process or dealing with cancer.

#### Usual care control

The UC control condition consists of patients receiving the expected, routine supportive care that adults with metastatic cancer would typically receive at RMCC. This includes the option to meet with the onsite clinical social worker, advanced practice provider, or oncologist, to discuss ACP, or to participate in other cancer support groups offered at RMCC or the broader community. To prevent diffusion of the intervention, the RMCC social workers are instructed to only use the M-ACT content with intervention participants. To ensure that all study participants have an opportunity to participate in the intervention, those randomized to UC are offered the opportunity to participate in a future M-ACT group upon completion of the final assessment (e.g., after the 2 month FU).

### Intervention training and facilitation

The M-ACT group sessions are co-led by two RMCC clinical social workers who were interested in learning ACT or participating in the study. Initial groups were led by J.L.M., an experienced ACT group facilitator at RMCC for eight years, and an additional social worker who was new to ACT. After these two social workers acquired initial experience leading the group, they and J.J.A. trained the remaining interested social workers in the ACT approach and the M-ACT protocol over multiple online sessions and a one-day workshop. The online and in-person trainings included active role-playing, experiential exercises, and coaching, reflecting evidence-based training approaches [53]. Throughout the study, J.J.A. and J.L.M. will provide weekly supervision and check-ins among the social workers currently leading the group sessions. In addition, 15 to 20% of M-ACT group sessions will be randomly selected for content fidelity ratings by ACT-trained doctoral students who are not involved in managing the study.

### Study ethics and integrity

The University of Colorado Boulder serves as the single IRB for the study. The Data Safety and Monitoring Committee at the University of Colorado Cancer Center provides additional oversight for data quality and participant safety and approves the study protocol and modifications. Written informed consent is obtained from all study participants preceded by a verbal discussion of the consent form by phone with one of the study coordinators to ensure full understanding and engagement with the consent process prior to signing. The CONSERVE-SPIRIT Extension guidelines [3] inform the trial design and reporting herein (see Table 2). Study coordinators randomize participants using a sequence created by study biostatistician S.J.S. via the embedded randomization function in REDCap wherein the sequence is hidden from the coordinator to reduce potential bias and once assigned cannot be altered. Measures are administered via REDCap by a member of the research team who is unaware of study condition. The confidentiality of data is ensured by following strict guidelines approved by the University of Colorado Boulder’s Office of Information Security (OIS), which oversees data security practices applied to all University research, on the collection, storage, and dissemination of data. All research team members complete relevant Collaborative Institutional Training Initiative (CITI) training and all university-affiliated members report an annual Disclosure of External Professional Activities (DEPA).

### Study timeline

As presented in Table 2, the primary and secondary outcomes and process variables are assessed at four timepoints: Pre, Mid (after the 3rd group session, with parallel timing in UC), Post (~3 days after the last weekly group session, with parallel timing in UC), and FU (2 months after the last weekly group session), with online surveys administered in REDCap [45]. Participants are paid $25 per survey timepoint for completing the survey, plus as $5 bonus for completing the survey in a specified amount of time (typically 36–48 h upon the survey link being sent), and a $30 bonus for completing all 4 surveys.

The screening measure (below) is administered orally by phone during the initial eligibility screening. Participants are enrolled up to six weeks before the weekly group intervention starts and completed the Pre measures generally 1 to 2 weeks prior to randomization to condition. After randomization, they continue through the 5 weeks of the weekly group intervention (with parallel timing in UC) and 2 months of post-intervention FU, for a total of 3.5 to 4 months time in the study.

### Measures

#### Screening measure

To be study-eligible, potential participants must score above the evidence-based cutoff of ≥ 3 on either the anxiety or depression scales of the Patient Health Questionnaire-4 (PHQ-4; [54, 55]) or, to account for the increased burden of comorbidity between anxiety and depression symptoms, a score of 4 or above on the total PHQ-4.

#### Primary outcome

The primary outcome is defined as an increase (from Pre) in the number of steps taken in the ACP process, which will be assessed with a checklist developed in the M-ACT pilot study. The checklist was developed in consultation with the Hospice and Palliative Nurses Association online ACP resources [50] as well as the study team’s experts in palliative care nursing and medicine, medical oncology, oncology social work, and biostatistics, and refined by soliciting pilot participants’ feedback on item clarity. The checklist describes each ACP step and asks patients to indicate any steps taken to date. Sample items include: “Have you thought about your values, goals, and preferences for care at the end of life?” or “Have you identified a health care decision maker?” The latter item includes a description of what this is, followed by items probing whether they discussed their values, goals, and decisions for end-of-life care with (each) their health care decision maker, oncology care team, family [or close friends (as desired)], or other health care providers. Additional items probe documentation, including whether they documented their health care decision maker using an official form (from among a list of legally accepted forms) and their “specific wishes for care at the end of life” using an official form (from among a list of legally accepted forms). Finally, the checklist asks whether they have given a copy of their directives to (each) their health care decision maker(s), oncology care team, family [or close friends], or any other health care providers. Reflecting the diversity of individual circumstance, patients are not required to complete ACP steps in a specific order. The steps most emphasized in the ACP checklist – reflecting on values and goals for end-of-life care, identifying and appointing a health care decision maker, and discussing values and goals for end of life care with one’s healthcare decision maker and health care team(s) (comprising 4 of 6 categories on the checklist) – are the ones most strongly emphasized in the M-ACT intervention.

Feedback from the online M-ACT pilot study resulted in two versions of this checklist, one for use at baseline (“have you ever…”) and one for use at each survey timepoint thereafter (“since *after* you began this study, please indicate which of the steps you’ve: -taken *for the first time* -*updated* - or discussed or done *again* since starting the study (if any)”) The instructions acknowledge that ACP is a process [12], preferences often evolve over time in the context of life-limiting disease, and the main goal of the study is to engage participants in the *process* of considering their values and goals for end of life care, discussing them with key others, and appointing and communicating with a health care decision maker. As an outcome variable, ACP process is operationalized as a count of steps taken or updated at each measurement wave.

#### Secondary outcomes

##### Depression and anxiety

The widely-used and validated *PHQ-8* and *GAD-7*, are used to assess depression and anxiety symptoms, respectively. In a large meta-analysis primarily for studies of diverse health care patients, the PHQ-8 evidenced good sensitivity and specificity for detecting major depressive disorder, with similar psychometric qualities as the PHQ-9 [56]. Among adults with various forms of cancer, the PHQ-9 shows good internal consistency α ≥ 0.84 [57]. Among primary care patients, the GAD-7 has an α = 0.82, test-retest reliability intraclass correlation (ICC) of 0.83, strong sensitivity and specificity, with increasing scores strongly associated with multiple domains of functional impairment [58].

##### Sense of life meaning

The *Functional Assessment of Chronic Illness Therapy Spiritual Well-Being Scale*, meaning/peace subscale [59] assesses sense of life meaning. Among cancer populations, this validated measure shows good psychometrics (α = 0.84-0.87, good convergent and divergent validity) and has been widely employed (e.g., [60, 61]) to assess a sense of meaning and purpose that is not specific to any particular religious or spiritual orientation.

##### Fear of death and dying

The *Death Attitude Profile Revised, Fear of Death and Death Avoidance Scales* [62, 63] is used to assess fear of death/dying. Among general adult and hospital/hospice nurse provider samples, this measure has good internal consistency (α = 0.82-0.88), acceptable test-retest reliability (*r* = .61-0.71), and good concurrent and discriminant validity.

#### Process measures

Core theorized ACT processes are evaluated using three brief measures with good psychometric properties. To assess acceptance/ defusion, we use the *Experiences Questionnaire*-Decentering scale [64], which shows strong psychometrics including α = 0.83-0.89, high test-retest reliability of *r* = .88 [65], good convergent and discriminant validity [64], and sensitivity to change [65]. To assess values-aligned behavior, we use the *Valuing Questionnaire* [66], which shows good convergent, discriminant, and incremental validity, and high internal validity (α = 0.87). To assess avoidance of feelings, a form of experiential avoidance, we use the *Multidimensional Experiential Avoidance Scale: Denial and Distress Avoidance Scales* [67], which also show good convergent, discriminant, and incremental validity and high internal validity (α = 0.82-0.89).

#### Intervention acceptability

Three indices are employed to evaluate intervention acceptability: (1) attendance in group sessions and completion of online modules and check-ins; (2) the widely-used and validated 8-item *Client Satisfaction Questionnaire* [68, 69], with item content adapted to the current study; (3) Likert ratings of the value of each group session on the piloted *Session Feedback Questionnaire* [32].

### Power and sample size estimation

Power calculations to evaluate group differences in outcomes over time Pre thru FU (Study Aim 1) were performed using the Optimal Design For Multilevel and Longitudinal Research Software, Version 3.01 [70], where the effect size estimates consider the number of cohorts/intervention groups, the average sample size per group, and the intraclass correlation (ICC) assessing within-cohort similiarities. Due to expected attrition, power analyses were based on an expected *n* = 174 (of total *n* = 240) participants contributing data at the final FU assessment, though this number can be considered conservative due to intent to apply modern data techniques to include patients with partial data. Though the M-ACT pilot study showed an average ICC between intervention cohort groups of less than 0.01, we conservatively estimated minimum effect sizes detectable for different power specifications for ICCs ranging from 0.02 to as high as 0.08. These ICC estimates were based on in-person intervention delivery, where cohorts would be similar by clinic and/or geographic area. Given the current switch to the online-only format due to COVID-19, within-cohort similarities are expected to be further attenuated. Power was calculated to detect treatment effects in changes over time, i.e., the condition by time interaction. Assuming two-sided alpha of 0.05 and minimum power of 80%, the proposed sample size will be sufficient to detect an effect size of Cohen’s *d* = 0.26 (or larger) of the M-ACT intervention over the UC condition on condition differences in change over time across the four time points. For the primary outcome of ACP, assuming a similar standard deviation of ± 2.29 as observed in the M-ACT pilot trial, this power corresponds to detecting a small but clinically important increase of approximately 0.60 ACP items. If the detectable effect size increases due to higher than anticipated attrition or ICC values, increases as low as 0.80 ACP items completed will still be detectable.

To evaluate relationships between ACP and anxiety and depression symptoms (Study Aim 2), the expected sample size will provide 80% power to detect an association of anxiety or depression symptoms with ACP as low as *r* = .25, with a multivariable regression model able to detect an R-squared of 0.08, a medium effect size. For the exploratory mediational aim, simulation [71] shows we will have ≥ 80% power to detect mediated effects associated with moderate to large sized path coefficients (e.g., as low as 6-13% of variance explained), which results in an ability to detect mediated effects that are smaller than relationships observed in the M-ACT pilot trial [42].

### Data analytic approach

For evaluating condition differences in primary and secondary outcomes (Study Aim 1), the analyses will focus on trajectories of change in primary and secondary outcomes over time and the impact of condition on these trajectories (i.e., time by condition interaction). Models will be appropriately developed in accordance with the distributional properties of each outcome. Analyses will utilize the four timepoints (Pre, Mid, Post, FU) and will involve the estimation of a series of latent growth curve models (LGM) [72] in MPlus [73] to test for differences between conditions on trajectories over the four time points. Unconditional growth models examining change in outcomes over time without condition or other fixed effects (e.g., gender) will be estimated first in order to assess the functional form of change (i.e., linear, quadratic).

For evaluating the relationships between ACP and anxiety/depression symptoms (Study Aim 2), we will use linear regression (for cross-sectional baseline data) and a structural equation modeling framework (for longitudinal data). For the baseline data, regression models will test the relationship of anxiety and depression symptoms each with ACP. After assessing for multicollinearity, multivariable models will be fit to examine anxiety and depression together. For the longitudinal data, we will test the hypothesis that decreases in anxiety and/or depression symptoms will be associated with increases in ACP over time. Parallel process growth models will be estimated in a structural equation modeling framework, using all timepoints. We will evaluate the relationship between the slopes, testing whether earlier change in anxiety or depression symptoms predicts subsequent change in ACP.

For the exploratory mediational aim, analyses will be carried out following established statistical methods for theoretical model testing with longitudinal data [74] and the potential for multiple mediators [75]. Specifically, we will use parallel process growth models to estimate change in mediators over time, using the product of coefficients method to compute the mediated effect and bias-corrected bootstrapped standard error estimates for evaluating statistical significance. Consistent with the current recommendations [76, 77], if Aim 1 is not supported, this will not affect the exploratory analyses of mediators as there is utility in examining putative mediators to inform potential mechanisms when designing future interventions.

### Data and safety monitoring and data sharing

A Safety Monitoring Committee helps monitor the trial, in addition to the University of Colorado Boulder IRB, PI (J.J.A.), and senior leadership team (R.M.F., S.J.S., D.J.A., J.L.M., J.S.K.). This committee consists of a medical oncologist and a clinical health psychologist who is a psycho-oncology researcher; both are independent of the study. The committee is consulted on an as-needed basis on matters of participant safety, adverse events, and protocol modifications. The committee receives an annual report on data integrity, adverse events and participant safety, with additional follow-up discussions conducted as needed.

Study findings will be published in peer-reviewed journals. The data will be shared with other scientists under the auspices of the PI unless requests become numerous, in which case it will be transferred to a data enclave. The PI, study coordinators, and study biostatistician will ensure that data are clearly documented (variable codebook, variable labels, etc.) for ease of use.

## Discussion

This RCT is evaluating a piloted, remotely-delivered, multi-modal palliative care intervention for anxious and depressed adults with metastatic cancer, with the group component of the intervention led by clinical social workers within community oncology clinics. The intervention targets adults across solid tumor advanced cancer type, using an evidence-based intervention approach (ACT) that addresses psychological symptoms and existential concerns, delivered within the context of a community cancer care setting. If successful, this study will make the following contributions: (1) By appropriately powering the study, we will demonstrate whether M-ACT benefits anxious and depressed adults with advanced cancer over usual care, informing the degree to which M-ACT is worthy of investing future resources; (2) By conducting the study in the community oncology setting with groups led by social worker providers, we will demonstrate whether the benefits of M-ACT are achievable in the setting where the most cancer patients receive care; (3) By shifting M-ACT to entirely remote delivery, this study evaluates an intervention delivered in a scalable manner that reduces study burden on participants; (4) By assessing M-ACT’s mechanisms of change, this study will explore which mechanisms drive outcomes—thereby elucidating how the intervention works; (5) By evaluating the relationships between anxiety/ depression symptoms and ACP, the study will inform whether such symptoms should be treated simultaneously with engaging patients in ACP and, if replicated, could create a new palliative care guideline. Given the high level of unmet palliative care needs in cancer and beyond [35, 36] and the broader need for palliative care interventions that do not require palliative care specialists and address multiple unmet needs in a single intervention, this study has the potential to advance the field while providing enduring benefits to adults with cancer.

## Data Availability

Not applicable.

## Abbreviations

ACP: Advance care planning
ACT: Acceptance and commitment therapy
M-ACT: Multi-modal acceptance and commitment therapy
PHQ: Patient health questionnaire
RCT: Randomized controlled trial
ICC: Intraclass correlation
IRB: Institutional review board
